# Ovarian hyperstimulation syndrome following surgical removal of a complete hydatidiform mole: a case report

**DOI:** 10.1186/s13256-019-2181-x

**Published:** 2019-09-11

**Authors:** Hiroaki Tsubokura, Yohei Ikoma, Takuya Yokoe, Tomoo Yoshimura, Katsuhiko Yasuda

**Affiliations:** 1 Department of Obstetrics and Gynecology, Kansai Medical University, 2-1-1 Sinmachi, Hirakata, Osaka 573-1010 Japan; 2 Department of Obstetrics and Gynecology, Kansai Medical University Medical Center, 10-15 Fumizono-cho, Moriguchi, Osaka 570-8507 Japan

**Keywords:** Complete hydatidiform mole, Magnetic resonance imaging, Ovarian hyperstimulation syndrome, Surgical removal

## Abstract

**Background:**

Generally, ovarian hyperstimulation syndrome develops after superovulation caused by ovulation-inducing drugs in infertile patients. However, ovarian hyperstimulation syndrome associated with natural pregnancy is rare, and most cases of ovarian hyperstimulation syndrome have been associated with a hydatidiform mole.

**Case presentation:**

We describe a case of a 16-year-old Japanese girl with a complete hydatidiform mole. The patient was referred for intensive examination and treatment of the hydatidiform mole and underwent surgical removal of the hydatidiform mole at 9 weeks, 5 days of gestation. Histopathological examination revealed a complete hydatidiform mole. The patient’s blood human chorionic gonadotropin level decreased from 980,823 IU/L to 44,815 IU/L on postoperative day 4, and it was below the cutoff level on postoperative day 64. Transvaginal ultrasonography on postoperative day 7 revealed a multilocular cyst measuring 82 × 43 mm in the right ovary and a multilocular cyst measuring 66 × 50 mm in the left ovary. Both ovarian cysts enlarged further. Magnetic resonance imaging on postoperative day 24 revealed that the right multilocular ovarian cyst had enlarged to 10 × 12 cm and that the left multilocular ovarian cyst had enlarged to 25 × 11 cm. Blood examination showed an elevated estradiol level as high as 3482 pg/ml. We diagnosed the patient with bilateral giant multilocular cysts accompanied by ovarian hyperstimulation syndrome because of the rapid increase in the size of the cysts. The patient complained of mild abdominal bloating; however, symptoms such as nausea, vomiting, dyspnea, and abdominal pain were not observed. Therefore, we chose spontaneous observation in the outpatient clinic. The cysts gradually decreased and disappeared on postoperative day 242.

**Conclusion:**

Physicians should be aware that ovarian cysts can occur and can increase rapidly after abortion of a hydatidiform mole. However, the ovarian cyst can return to its original size spontaneously even if it becomes huge.

## Background

Ovarian hyperstimulation syndrome (OHSS) commonly develops after superovulation caused by ovulation-inducing drugs in infertile patients. Ninety percent of patients have mild OHSS that improves with only follow-up observation. However, symptomatic therapy is necessary for moderate or severe cases, and in severe cases, there is a possibility of death because of thrombus or embolism. OHSS associated with natural pregnancy is rare, and so far, most cases of OHSS have been associated with a hydatidiform mole. OHSS manifests with bilateral ovarian enlargement in addition to symptoms such as ascites or pleural effusion caused by hypervascular permeability, hemoconcentration, and oliguria. In this report, we describe a patient in whom a giant ovarian cyst measuring 25 cm appeared suddenly after surgical removal of a complete hydatidiform mole and returned to normal ovarian size during the follow-up observation period without treatment.

## Case presentation

A Japanese girl aged 16 years, 7 months, gravida 1 para 0, was referred to our hospital because of a suspicious complete hydatidiform mole. Her height was 152 cm, weight was 40.8 kg, and body mass index (BMI) was 17.7 kg/m^2^. Her blood pressure and heart rate were 110/60 mmHg and 88/minute, respectively. Her age at menarche was 13 years, her menstrual cycle was 30 days, and her periods lasted 5 days. The patient had undergone surgery for choledochal dilation at 2 years of age and had no significant family history. She was diagnosed with a complete hydatidiform mole at 9 weeks, 0 days of gestation from the last menstruation.

A small amount of dark red genital bleeding was observed during the medical examination, and the uterus was the size of a neonatal head, which was larger than the size corresponding to 9 weeks, 0 days of gestation. Transvaginal ultrasonography did not show a gestational sac and embryo, but it revealed many small cysts in the uterus. No bilateral ovarian swelling was observed. The blood human chorionic gonadotropin (hCG) level was markedly increased and was as high as 980,823 IU (normal limit < 5 IU). On the basis of these findings, we diagnosed her pregnancy as a complete hydatidiform mole, and legally induced abortion was performed at 9 weeks, 5 days of gestation using an aspiration instrument. We explained the necessity of the procedure to the patient and her parents and obtained written informed consent from them.

Macroscopically, the uterine content was only cystic villi without obvious fetal components. Histopathological examination also revealed a complete hydatidiform mole. The patient’s blood hCG level decreased to 44,815 IU/L on postoperative day 4 and to 120 IU/L on postoperative day 29, and it was below the cutoff level on postoperative day 64. Additionally, menstruation occurred spontaneously on postoperative day 32.

**Fig. 1:**
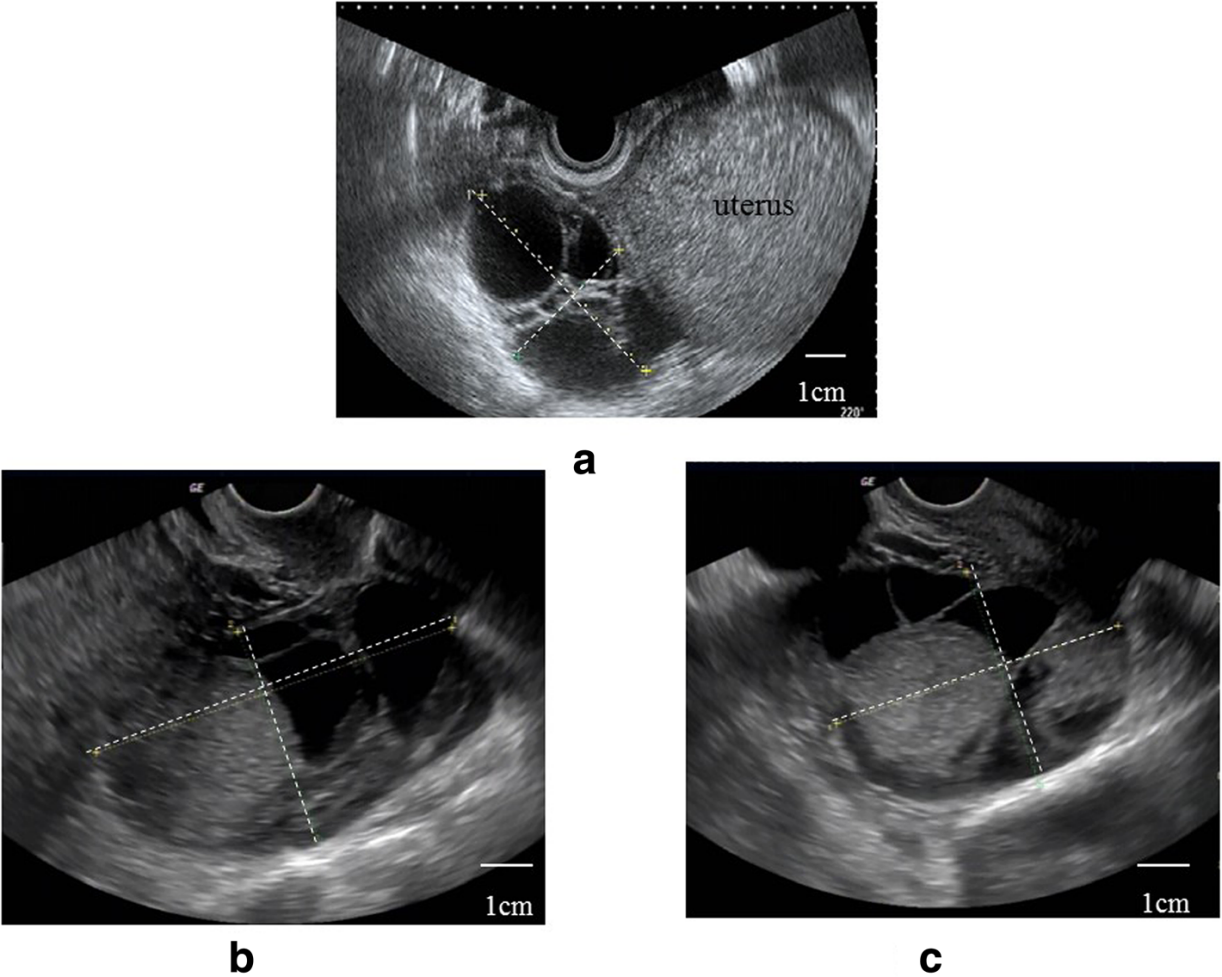
Transvaginal ultrasonograms of the ovarian cysts. **a** A 71 × 43–mm right ovarian cyst on postoperative day 4. **b** A 82 × 43–mm right ovarian cyst on postoperative day 7. **c** A 66 × 50–mm left ovarian cyst on postoperative day 7

**Fig. 2:**
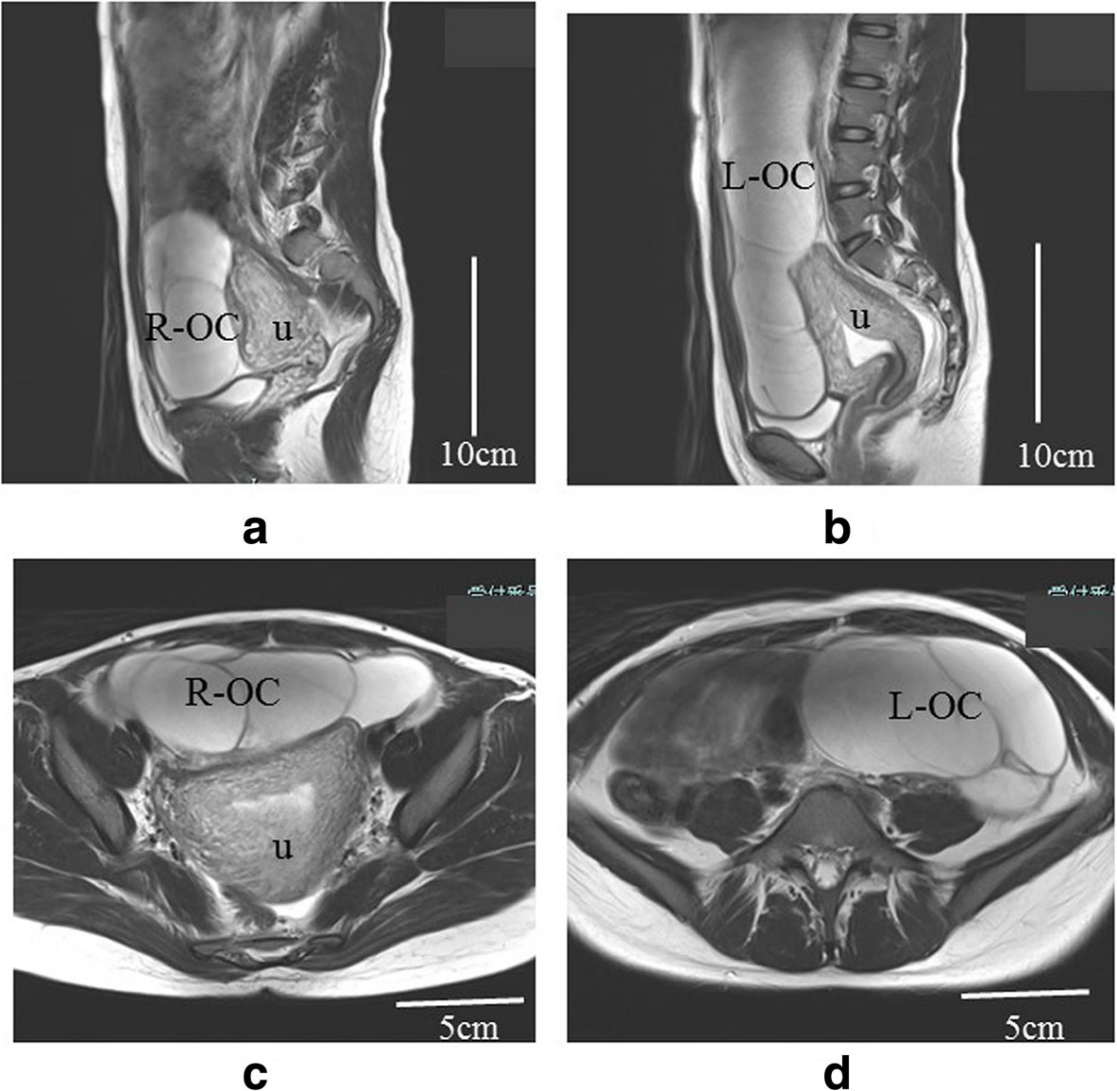
Magnetic resonance imaging scans of the ovarian cysts on postoperative day 24. **a** Sagittal view of the right ovarian cyst (R-OC). **b** Sagittal view of the left ovarian cyst (L-OC). **c** Transverse view of the R-OC. **d** Transverse view of the L-OC. u, uterus

Transvaginal ultrasonography revealed a multilocular cyst measuring 71 × 43 mm in the right ovary on postoperative day 4, and the cyst increased to 82 × 43 mm on postoperative day 7 (Fig. 1a, b). No swelling was observed in the left ovary on postoperative day 4, but a multilocular cyst measuring 66 × 50 mm was observed on postoperative day 7 (Fig. 1c). The bilateral ovarian cysts enlarged further; magnetic resonance imaging on postoperative day 24 revealed that the right multilocular ovarian cyst had enlarged to 10 × 12 cm and that the left multilocular ovarian cyst had enlarged to 25 × 11 cm (Fig. 2a–d). Additionally, a small amount of ascites was recognized. The patient complained of mild abdominal bloating, but no symptoms such as nausea, vomiting, dyspnea, and abdominal pain were observed. Blood examination showed elevated E2 as high as 3482 pg/ml. However, hemoconcentration, electrolyte abnormalities, hypoalbuminemia, elevated liver enzyme levels, and renal dysfunction were not observed. Regarding blood tumor markers, only the cancer antigen 125 (CA 125) level was elevated, to 134.7 U/ml, but the α-fetoprotein, carcinoembryonic antigen, sialyl Tn antigen, and carbohydrate antigen 19-9 levels were within normal limits. A giant ovarian cyst with a high CA 125 level and a small amount of ascites are symptoms that mimic a malignant ovarian tumor. Therefore, we first considered an operation for the ovarian cyst. However, enlargement of the ovarian cyst was too rapid, even though the cyst might have been a malignant tumor. Additionally, we found few case reports describing an ovarian cyst accompanied by OHSS following hydatidiform mole in the literature. Therefore, we diagnosed the disease as bilateral giant multilocular cysts accompanied by OHSS following surgical removal of a complete hydatidiform mole. We also diagnosed the cause of the elevated CA 125 level as ascitic fluid accumulation associated with OHSS. Therefore, we opted for spontaneous observation in the outpatient clinic and expected the bilateral ovarian cysts to decrease in size.

**Fig. 3:**
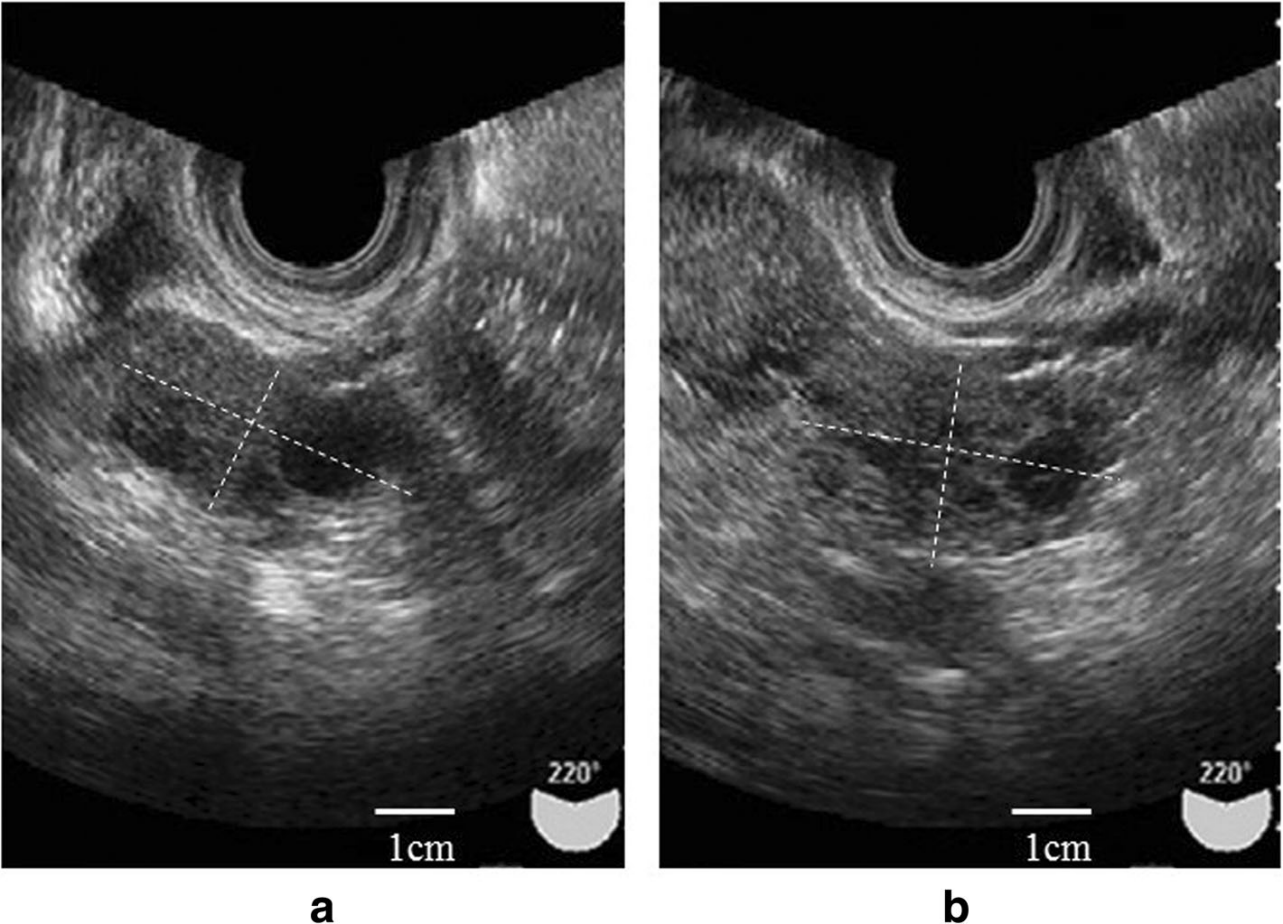
Transvaginal ultrasonograms on postoperative day 242. **a** Restored right ovary. **b** Restored left ovary

As expected, the size of the cysts decreased gradually during the observation period, and we confirmed their disappearance on postoperative day 242 (Fig. 3a, b). The patient is still under observation, and we have confirmed the absence of ovarian cysts.

## Discussion

Generally, OHSS is known as an iatrogenic disease caused by ovarian stimulation performed as a type of infertility treatment. Excessive ovarian stimulation causes multilocular ovarian cyst formation, dehydration, hemoconcentration, storage of ascites or pleural effusion, dyspnea, oliguria, and electrolyte abnormality (hyponatremia and hyperkalemia). Then hypotension, tachycardia, and decreased central venous pressure develop, and finally a critical event occurs [1]. The occurrence of OHSS accompanied by spontaneous pregnancy is rare. As shown in Table 1, only 11 cases of OHSS after spontaneous pregnancy, with abnormal chorionic villi, have been reported until now, and of those, 9 cases were associated with a hydatidiform mole, one with an invasive mole, and one with placental mesenchymal dysplasia [2–12]. Among nine cases of OHSS that occurred after abortion of a hydatidiform mole, the first case was reported in 1966, and the ninth case was reported in 2015. Thus, nine cases of OHSS following hydatidiform mole were reported within a span of about 50 years (Table 1).

**Table 1:** Summary of reported cases of ovarian hyperstimulation syndrome accompanied by chorionic diseases

References	Age (years)	Gestation (weeks)	Gravida and para status	Pathology	Outcome of pregnancy	hCG before treatment for chorionic disease	hCG at occurrence of OHSS	Occurrence time after treatment	Maximum size of enlarged ovary	Massive ascites	Pleural effusion	Main treatment for OHSS	Duration of hospitalization	Duration of recovery to normal-sized ovary
Hooper et al. [2]	25	7	G0P0	Mole (type, not described)	Spontaneous abortion	Not described	Not described	7 days	Right: 17.5 cm Left: 12.5 cm	+	Not described	Paracentesis, right salpingo-oophorectomy, left wedge resection	Not described	6 weeks
Moneta et al. [3]	25	16	G2P0	Mole (type, not described)	Legal induced abortion	280,000 IU/24 h	About 5000 IU/24 h	8 days	Right: about 10 cm Left: about 10 cm	+	+	Paracentesis, infusion, albumin, blood transfusion	1 month	Not described
Ludwig et al. [4]	Not described	14	G3P1	Partial hydatidiform mole	Legal induced abortion	1350 IU/L	7320 IU/L	14 days	Right:17x10x15 cm Left:16x10x14 cm	+	+	Infusion, heparin	6 days	Not described
Arora et al. [5]	23	12	G3P2	Partial hydatidiform mole	Legal induced abortion	400,000 IU/L	65,554 IU/L	3 days	Right:11 × 11.2 cm Left:13.6 × 12.8 cm	+	+	Paracentesis, infusion	11 days	Not described
Strafford et al. [6]	19	12	G1P0	Complete hydatidiform mole	Legal induced abortion	811,506 IU/L	23,681 IU/L	6 days	Right:12x8x7 cm Left:9x8x11 cm	+	+	Infusion, heparin, pleural drainage	20 days	3 months
Rachad et al. [7]	34	12	G4P3	Invasive mole	Ovarian drilling followed by chemotherapy	2,000,000 IU/L	2,000,000 IU/L	0	Right:16 cm Left:12 cm	+	+	Infusion, albumin, heparin, chemotherapy, paracentesis, hysterectomy	2 months	2 months
Zhou et al. [8]	38	16	G5P4	Complete hydatidiform mole	Legal induced abortion	860,000 IU/L	45,674 IU/L	7 days	Right: 12.1 cm Left: 11.7 cm	+	+	Paracentesis, thoracentesis, albumin	2 weeks	3 months
Diness et al. [9]	29	15	Not described	Partial hydatidiform mole	Legal induced abortion	151,000 IU/L	5220 IU/L	3 days	Right:14 cm Left:14 cm	+	–	Infusion	9 days	4 months
Suzuki et al. [10]	31	10	G not described P2	Partial hydatidiform mole	Legal induced abortion	390,000 IU/L	9830 IU/L	8 days	30 cm (not described about right or left)	+	+	Not described	9 days	1 month
Wu et al. [11]	29	10	G1P0	Partial hydatidiform mole followed by invasive mole	Legal induced abortion followed by chemotherapy	225,000 IU/L	67,800 IU/L	19 days	Right:17.2 × 15.9 × 17.8 cm Left:11.4 × 18.1 × 17.7 cm	+	+	Infusion, albumin, heparin, chemotherapy	A few days	7 months
Davoudian et al. [12]	20	16	G1P0	Placental mesenchymal dysplasia	Spontaneous abortion	Not described	24,890 IU/L	Not described	Right: 10 cm Left: 9 cm	–	–	–	0	A few weeks
Our patient	16	12	G1P0	Complete hydatidiform mole	Legal induced abortion	980,823 IU/L	44,815 IU/L	4 days	Right:10 × 12 cm Left:25 × 11 cm	–	–	–	0	8 months

*hCG* Human chorionic gonadotropin, *OHSS* Ovarian hyperstimulation syndrome

OHSS is classified as mild, moderate, or severe depending on the clinical symptoms and blood test findings. Most cases of OHSS are classified as mild, but 1–5% of cases are classified as moderate or severe [13]. In mild cases, spontaneous recovery is observed during the observation period, but in moderate or severe cases, appropriate symptomatic treatments are necessary [14]. In previously reported cases (Table 1), OHSS occurred after abortion of the hydatidiform mole; all patients were hospitalized; and the patients received treatments such as fluid infusion and albumin administration, heparin administration to prevent thrombosis or embolism, electrolyte replacement, and puncture or aspiration of ascites or pleural effusion. However, in our patient, neither severe clinical symptoms, such as massive ascites or pleural effusion, nor abnormal blood test results were found. Thus, neither hospitalization nor symptomatic treatment was required, although the patient had a huge ovarian mass. The absence of ascites or pleural effusion may have been because of the patient’s small and thin structure (BMI 17.7 kg/m^2^). Therefore, the intra-abdominal pressure of the patient was expected to be extremely high because the huge ovarian cyst occupied the abdominal cavity, and the leakage from the blood vessel may have been suppressed, preventing severe clinical symptoms.

Most cases of iatrogenic OHSS are caused by the administration of hCG, and the symptoms worsen with pregnancy and improve after abortion. In the above-referenced cases of OHSS associated with a hydatidiform mole, however, the onset of OHSS was after abortion, and the symptoms worsened. In all cases, OHSS developed when the serum hCG level was low, after induced or spontaneous abortions, and not when the serum hCG level was the highest. Regarding the discrepancy, Ludwig *et al.* pointed out the involvement of vascular endothelial growth factor (VEGF) in the onset of OHSS because the serum VEGF concentration was high at the onset of OHSS in their patient’s case [4]. However, Strafford et al. reported that the serum VEGF concentration did not increase at the onset of OHSS but that the serum E2 concentration increased [6]. In our patient, the serum E2 concentration was markedly high, even though induced abortion was performed. Our patient’s case also follows the same process as that reported by Strafford et al. [6]. However, De Leener et al. reported that a mutation of the follicle-stimulating hormone (FSH) receptor was found in patients who developed OHSS, although the serum hCG concentration was within the normal limit [15]. Mutation of the FSH receptor was also reported by Wu et al. [11]. Thus, the onset of OHSS may be due to the effects of some factors such as the hCG level, E2 level, and abnormal FSH receptor.

According to the literature, the 25-cm ovarian cyst derived from the left ovary in our patient might be the second largest cyst among ovarian cysts accompanied by OHSS. The largest ovarian cyst was 30 cm, which was reported by Suzuki et al. [10]. Unfortunately, the authors did not show any image of the 30-cm ovarian cyst or describe whether the ovarian cyst was derived from the left ovary or the right ovary. In our patient, we measured the size of the ovarian cyst by using magnetic resonance imaging (MRI) because transvaginal and abdominal ultrasonography could not reveal the size of the ovarian cyst. Additionally, MRI was convenient for distinguishing between the left cyst and the right cyst.

The duration for reduction of the size of multilocular ovarian cysts in OHSS to that of a normal ovary is reported to range from 1 month to 7 months. Surprisingly, Suzuki et al. reported that a 30-cm ovarian cyst returned to its original size within only 1 month [10]. Conversely, Wu et al. reported that it took 7 months for an 18-cm ovarian cyst to return to its original size [11]. In our patient, it took 8 months for the 25-cm ovarian cyst to return to its original size. It seems that the duration required for reduction of the cyst does not depend on the size of the ovarian cyst. These results suggest that ovarian cysts in OHSS consistently return to their original size, although they may be huge and the duration of reduction in size is long. Additionally, surgical procedures such as ovarian cystectomy and ovarian drilling may be unnecessary.

## Conclusions

OHSS associated with natural pregnancy is rare, but most cases of OHSS have been associated with a hydatidiform mole. Therefore, physicians should be aware that ovarian cysts accompanied by OHSS can occur after abortion of a hydatidiform mole and that the size of the ovarian cyst can increase rapidly, although this is rare. Additionally, there is a reported case of a giant ovarian cyst with few symptoms of OHSS; most similar cases have many symptoms, including massive ascites and pleural effusion. However, most ovarian cysts return spontaneously to their original size after several months, even the huge ones.

### Patient perspective

The patient understood why both her ovaries became enlarged after the legally induced abortion for a complete hydatidiform mole, and she hopes that ovarian enlargement will never recur.

## Data Availability

The datasets used and/or analyzed in the current study are available from the corresponding author upon reasonable request.

## Abbreviations

BMI: Body mass index
CA 125: Cancer antigen 125
FSH: Follicle-stimulating hormone
hCG: Human chorionic gonadotropin
MRI: Magnetic resonance imaging
OHSS: Ovarian hyperstimulation syndrome
VEGF: Vascular endothelial growth factor
